# 3640 Unique EST Clusters from the Medaka Testis and Their Potential Use for Identifying Conserved Testicular Gene Expression in Fish and Mammals

**DOI:** 10.1371/journal.pone.0003915

**Published:** 2008-12-23

**Authors:** Lijan Lo, Zhenhai Zhang, Ni Hong, Jinrong Peng, Yunhan Hong

**Affiliations:** 1 Department of Biology Sciences, National University of Singapore, National University of Singapore, Singapore, Singapore; 2 Institute of Molecular and Cell Biology, Proteos, Singapore; National Institutes of Health, United States of America

## Abstract

**Background:**

The fish medaka is the first vertebrate capable of full spermatogenesis in vitro from self-renewing spermatogonial stem cells to motile test-tube sperm. Precise staging and molecular dissection of this process has been hampered by the lack of suitable molecular markers.

**Methodology and Principal Findings:**

We have generated a normalized medaka testis cDNA library and obtained 7040 high quality sequences representing 3641 unique gene clusters. Among these, 1197 unique clusters are homologous to known genes, and 2444 appear to be novel genes. Ontology analysis shows that the 1197 gene products are implicated in diverse molecular and cellular processes. These genes include markers for all major types of testicular somatic and germ cells. Furthermore, markers were identified for major spermatogenic stages ranging from spermatogonial stem cell self-renewal to meiosis entry, progression and completion. Intriguingly, the medaka testis expresses at least 13 homologs of the 33 mouse X-chromosomal genes that are enriched in the testis. More importantly, we show that key components of several signaling pathways known to be important for testicular function in mammals are well represented in the medaka testicular EST collection.

**Conclusions/Significance:**

Medaka exhibits a considerable similarity in testicular gene expression to mammals. The medaka testicular EST collection we obtained has wide range coverage and will not only consolidate our knowledge on the comparative analysis of known genes' functions in the testis but also provide a rich resource to dissect molecular events and mechanism of spermatogenesis in vivo and in vitro in medaka as an excellent vertebrate model.

## Introduction

The testis is the male gonad where spermatogenesis takes place throughout adult life to continuously supply sperm for the next generation. Defects in testicular structure and function lead to testicular tumors and male infertility. In mammals, the adult testis consists of male germ cells and three major somatic cell types [1]. The germ cells undergo spermatogenesis through sequential stages that exhibit remarkably differential gene expression. The somatic cells are Sertoli, Leydig and peritubular myoid cells, which express different molecules and provide the environment to maintain sexual development, support and orchestrate spermatogenesis.

Much is known about the cell biology of spermatogenic germ cell development, which proceeds through three major stages: mitotic phase of proliferation and differentiation, meiosis and postmeiotic spermiogenesis [1]. Meiosis results in round spermatids, spermiogenesis leads to sperm. In mammals, in vitro spermatogenesis cannot proceed beyond the spermatid stage [2], [3]. In lower vertebrates like fish, however, in vitro spermatogenesis from spermatocytes can proceed fully to produce fertile sperm [4], [5]. Specifically, medaka spermatocytes in culture can give rise to functional sperm without any supporting cells. Previously we have established that the medaka fish is a unique vertebrate model for the in vitro recapitulation of full spermatogenesis from a self-renewing spermatogonial cell line through meiosis to motile sperm [6]. However, the analysis of molecular events and mechanism of medaka spermatogenesis in vitro and in vivo has been hindered in this organism by the paucity of suitable molecular markers for various types of cells at different stages. One of the approaches is to obtain the testicular transcriptome or expressed sequence tags (ESTs). Testicular transcriptome or EST projects have recently been reported in mouse [7]–[11] and human [12]. In fish, testicular EST collections have been reported in two species. Zeng and Gong [13] reported 501 testicular ESTs in zebrafish, and Chini et al [14] described 2907 ESTs for the blue fin tuna testis.

This study aimed to establish a medaka testicular EST collection. For this, we generated a normalized cDNA library from the adult medaka testis and sequenced 7040 random EST clones. Comparative sequence analysis revealed a total of 3641 unique gene clusters.

## Results and Discussions

### Construction of a normalized medaka testicular cDNA library

**Figure 1:**
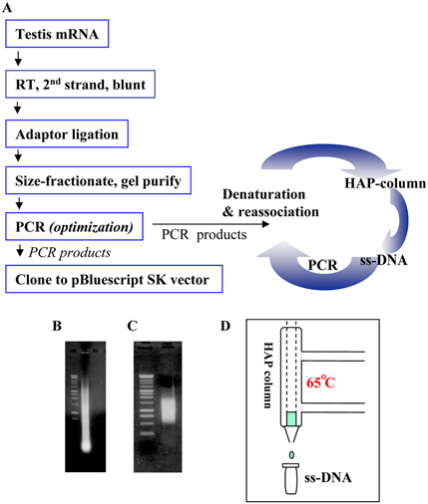
Construction of a normalized medaka testicular cDNA library. (A) Diagram showing the procedure for cDNA normalization. The cDNA library was constructed from cDNA after three rounds of normalization. RT: reverse transcription. (B and C) Size-selection of cDNA for normalization. Total cDNA before size-selection (B) and size-selected cDNA (C). (D) Sketch showing the setup of a jacketed HA-column. ss-DNA: single-stranded DNA.

The expression levels of each individual genes in a genome can vary considerably in different cells, tissues or organs, and can even be very different in the same tissue or organ at different developmental stages or physiological conditions. The magnitude of difference can range from a few to as much as thousand folds. When RNA samples from an organism or an organ or a tissue are directly used for cDNA library construction, the differences among different genes in expression levels will normally be reflected in such libraries. If such a library is used for EST sequencing project, the problem of high redundancy will be brought in [15], [16]. A common practice to avoid high redundancy during EST sequencing is to construct a normalized cDNA library. The principle for constructing normalized cDNA libraries is based on the fact that, during cDNA annealing, rare cDNA transcript anneal less rapidly than abundant cDNA species, thus the single-stranded fraction of cDNA (ss-cDNA) becomes progressively more normalized during the course of annealing [17], [18]. To reduce redundant sequencing, we constructed a normalized medaka testicular cDNA library. The procedure of normalization is illustrated in Figure 1A (for details, see Materials and Methods). In brief, total RNA was isolated from a pool of adult testes, mRNA was purified for cDNA synthesis. The resulting double stranded (ds) cDNAs were linked to an adaptor, and after fractionation, those between 0.5–2.0 kb were recovered (Figure 1B and 1C). Normalization of cDNAs was performed by three rounds of PCR – denaturation – reassociation – ss-cDNA purification – PCR ([17]; also see Materials and Methods). ss-cDNA was enriched and purified using hydroxyapatite chromatography (HA-column) (Figure 1D), which effectively separates the ss-cDNA from ds-cDNA. Finally, the normalized ss-cDNA was used as the template for the synthesis of ds-cDNA for the library construction.

### EST clones and sequencing

A total of 8736 clones from the normalized cDNA library was sequenced, generating 7040 high quality (7033 having reads ≥200 bp read each) EST sequences (EMBO Accession numbers FM165707–FM172746). These 7040 sequences were subjected to clustering analysis using CLUSTAL W (1.83) Multiple Sequence Alignments program and 3641 clusters were obtained (Table 1; Table S1). Overall redundancy is around 48% and this rate is significantly lower than that for a non-normalized zebrafish cDNA library [15]. Statistical analysis showed that 743 out of 3641 clusters are each represented by a single clone, 2450 clusters by two clones and the remaining 448 clusters by 3–6 clones (Table 2). There are only two clusters containing 6 individual clones each and these two clusters represent two novel genes and are the most abundant in this library. No cluster was found to contain more than 6 clones. This is in sharp contrast to the observed large number of redundant clones in non-normalized zebrafish and medaka cDNA libraries [15], [16]. In a separate EST sequencing effort, 747 ESTs were obtained from an unnormalized medaka testis cDNA library (Laszlo Orban and YHH, unpublished). Sequence analysis showed that *synaptonemal complex protein 3* (*SCP3*, meiosis marker) gene and *kallikreini* gene (Leydig cell marker) was represented by six and two EST clones, respectively, in the 747 unnormalized EST collection whereas no corresponding clones was found in our 7040 EST clones. *synaptonemal complex protein 1* gene (SCP1, meiosis marker) was represented by three EST clones in the 747 unnormalized ESTs and was represented by only one clone in our EST set. From the mathematic view, all these facts demonstrate the effectiveness of normalization in our library.

**Table 1:** Summary of statistics of ESTs obtained from medaka testis.

Total ESTs obtained	7040
Total unique clusters identified	3641
Clusters matching sequences in the medaka UniGene database	1034
Clusters having hits in the nr database	2057
Clusters having no hits in the nr database	550

**Table 2:** Clustering analysis of 7040 ESTs.

Number of EST clones in a cluster	1	2	3	4	5	6
Number of clusters	743	2450	403	39	4	2

The total EST collection for medaka in NCBI database is approaching 315,000 (http://www.ncbi.nlm.nih.gov/UniGene/; dated at February 28^th^, 2008) [16]. Clustering analysis of these ESTs identified a total of 17307 Unigene clusters as released on February 2008 (ftp://ftp.ncbi.nih.gov/repository/UniGene/Oryzias_latipes/). The longest sequences in each of our EST clusters were retrieved and used to blast search against the NCBI medaka Unigene database, with the aim to assess the representation of our library in the public database. Unexpectedly, only a small fraction of our 3641 unique clusters (n = 1034; 28%) exhibit a match in the NCBI medaka UniGene database (Table 1; Table S2), whereas the majority (n = 2607; 72%) do not hit, representing new gene clusters for medaka expressed genes. The addition of the medaka testicular ESTs we obtained to the existing database gives rise to a total of 19914 unique gene clusters for medaka and these ESTs are invaluable references in assisting the annotation of medaka genome in the near future. Due to the fact that some cDNAs may contain more than one *EcoRI* sites and that *EcoRI* fragments were used to construct the normalized cDNA library, the final number of unique clusters could be overestimated when fragments generated from the same cDNA failed to form one cluster due to sequence discontinuity.

### Characteristics of medaka testicular ESTs

For the 1034 of our EST clusters that have corresponding hits in the NCBI medaka Unigene database we downloaded the gene information for these Unigenes based on Unigene number (Table S2). The 2607 new unique clusters in our EST collection that have no matches in the NCBI medaka Unigene database their sequences were translated into all six frames to blast the NCBI blastx database (ftp://ftp.ncbi.nlm.nih.gov/blast/), and 2058 clusters were found to have their corresponding hits with high confidence in the non-redundant database (Table S3). Due to the fact that the size of cDNA was selected between 0.5–2.0 kb after cDNA was synthesized using oligo-dT, for those genes with long 3′-UTR, the sequences obtained might not have reached their ORF sequences. Therefore, it is possible that the rate of sequences with no hit in nr database is overestimated.

Analyzing the 1034 clusters that identified their corresponding Unigenes in the database and the 2058 clusters that have hits in the NCBI database revealed that 550/1034 Unigenes and genes corresponding to 647/2058 clusters have each been assigned a putative molecular function based on amino acid sequence homology, whereas the rest of Unigenes and gene clusters are recorded as hypothetical, unnamed or predicted genes (functionally unassigned putative genes) (Table S2 and S3).

The 1197 genes (550 Unigenes and genes corresponding to 647 clusters) with known putative molecular function were combined and subjected to ontology analysis based on molecular and cellular functions. As expected, genes encoding for metabolic enzymes (including hydroxylase, oxidase, reductase, dehydrogenase, synthase, metabolic kinase and phosphatase, transferase) form the major group in our EST set and in total 162 out of 1197 (∼14%) genes were recorded (Table 3, Table S4). A total of 63 out of 1197 genes are found to encode products related to DNA biosynthesis (e.g polymerase for replication and reverse transcriptase for reverse transcription) and to DNA structure and stability maintenance (e.g DNA binding protein, telomerase binding protein etc) while 60 genes are for RNA biosynthesis (polymerase), maturation (e.g ribonucleoprotein) and degradation (e.g RNase) and 100 genes for protein biosynthesis (e.g ribosomal proteins), protein conformation (e.g chaperones) and degradation (e.g proteins involved in proteasome pathway) (Table 3, Table S4). In total, 94 genes were found to encode proteins related to transcription regulation (including transcription factors and coactivators), 54 related to cellular motor complex, 41 related to exocytosis and endocytosis and 41 related to cell cycle and cell death (e.g apoptosis) (Table 3, Table S4). A significant number of genes encode products for extracellular proteins (52/1197) (including extracellular matrix protein and carrier etc), transmembrane proteins (excluding receptors) (49/1197) and proteins with binding activity (44/1197) (excluding receptors, carriers, and DNA and RNA binding proteins) (Table 3, Table S4). A group of genes (65/1197) are also linked to different diseases (causal factor or antigens) (Table 3, Table S4). Surprisingly, molecules involved in different signaling pathways (including different types of ligands, receptors, protein kinases and phosphatases, signal transducers and cofactors) constitute the largest group with a total 188 genes falling in this category (Table 3, Table S4). The remaining 174/1197 (∼14.5%) genes encode products involved in many other diverse biochemical and cellular processes (Table 3, Table S4).

**Table 3:** Summary of ontology analysis of 1197 genes with known putative functions.

Molecular and cellular process	Molecular function	Number of genes
Cellular signaling (total: 188)	Protein kinase	45
	receptor	41
	Others (ligand, cofactors, protein phosphatase, transducers etc)	102
Transcription regulation (total: 94)	Zinc finger	30
	Others	64
DNA biosynthesis, structure and stability maintenance (0total: 63)	Polymerase	6
	Transposase	13
	Others (DNA binding protein, replication complex, nucleosome assembly protein etc)	44
RNA biosynthesis, maturation and stability (total: 60)	Polymerase	10
	RNA binding protein	11
	Others (RNase, RNA helicase, RNA processing protein etc)	39
Protein biosynthesis, conformation and degradation (total: 100)	Ribosomal protein	20
	Chaperone	10
	Proteasome pathway	38
	Others (proteinase, tRNA synthase, translation initiation factor etc)	32
Cell cycle and cell death (total: 41)	Cell cycle	35
	Apoptosis	5
Extracellular protein (total: 52)	Extracellular matrix protein	26
	Carrier	14
	Others	12
Transmembrane and channel protein (total: 49)	Transmembrane protein	30
	Channel protein	19
Metabolic enzymes		162
Motor proteins		54
Trafficking		41
Disease-related		65
Protein with binding activity		44
WD repeat protein		8

It would be interesting to compare medaka testis-enriched genes with other vertebrates through genomic analysis. For example, are the genes conserved among the vertebrates? Is there any synteny relationship among these conserved genes? Are these genes alternatively spliced from annotated genes? Are there genes completely new without any annotated protein domains? However, these bioinformatic analyses rely on the availability of full length cDNA. This is mainly because cross-species comparison is normally performed on the amino acid sequence level but not at the nucleotide sequence level (because of the high variation of nucleotide sequences for ortholog genes between species during evolution). Use the amino acid sequences derived from full length cDNA will permit the identification of true conserved or novel genes. Then the in situ hybridization method can be used to prove if the novel gene is enriched in the testis. For this purpose, we designed a python program to retrieve information for the 1034 unigenes listed in Table S2 from NCBI. For each unigene cluster, the section ‘Sequences’ was screened for mRNA sequence information and mRNA sequence information (e.g gene identification (id) number, gene annotation, complete cDNA sequence (cds) or not) was retrieved and analyzed. For 1034 unigenes, only 48 of them have mRNA sequences information available in the unigene database and among which 28 of them have complete cds (Table S5). Apparently, the lack of full length cDNA sequences in medaka in general disallowed us to perform more detailed systematic bioinformatic analysis. This fact further shows the importance of our EST set for gene annotation in medaka in the future. In addition, we can get important information (though not authentic) from our EST analysis which will guide us to select a group of target genes for in situ hybridization to identify who are genuinely enriched in the testis. Based on the in situ hybridization result we then can get the full length sequences corresponding to these testis-enriched genes, then we can perform cross-species analysis to finally answer if it is a novel testis –enriched gene.

### Counterparts of well-known mammalian testicular genes in medaka

The availability of 1197 gene homologs in our medaka testicular EST collection enabled us to search for conserved testicular genes between fish and mammals. At least 50 genes whose mammalian homologs are highly expressed in the testis have been identified in the medaka testicular EST collection. These include genes encoding transcription regulators (*Blimp1*, *Bmi1*, *YY1* etc) and receptors, ligands and signaling molecules (*Notch*, *Lifr*, *BMPR1*, and *TGFR1* etc) in testis (Table 4) [19]–[28]. Gene markers for different cell types in testis and for different stages of spermatogenesis are also among the list, including somatic cell markers (*Dmrt1* and *cytochrome P450 11β* for Sertoli cell; *3-beta hydroxysteroid dehydrogenase* and *luteinizing hormone receptor/gonadotropin receptor II* for Leydig cell; *beta-catenin-binding protein* for myoid cell), germ cell markers (*Bruno2*, *Gasz*, *Gustavus*, *polo-like kinase*, *piwi* etc), pre-meiosis markers (*Cyclin E1*, *ALF*, *CDC-like 2* etc), meiosis regulator&structural protein (*Gld1*, *meiosis-activating kinase*, *SCP1*, *Rad51*, *NME2* etc), and postmeiosis marker (*Msap* and *Rsh*) (Table 5) [29].

**Table 4:** Signaling molecules and transcription regulators in the medaka testicular EST collection.

Gene name	Description	EST Clone ID
	***Receptors, ligands and signaling molecules***	
Notch1	Receptor for Notch signalling	M039–B1_013
Notch2	Receptor for Notch signalling	M071–B2_014
Notch3	Receptor for Notch signalling	m014–G1_003
Lifr	leukemia inhibitory factor receptor alpha	M036–H11_081
gp130	leukemia inhibitory factor receptor	M064–C3_027
Stam	signal transduction adaptor molecule	M030–H3_017
BMPR1a (Alk3)	Bone morphogenetic protein receptor 1a	M035–G10_068
Smad2/3	Component of BMP signalling pathway	M065–G10_068
Smad4	Component of BMP signalling pathway	M057–E7_055
Smad anchor	Smad anchor for receptor activation	M090–H2_002
TGFβR	TGFβ receptor	M063–A2_016
PI3K	phosphatidylinositol 3 kinase	M089–G8_052
Pten1	Phosphatase and tensin homolog, antagonist of PI3K	M043–G10_068
Akt/PKBβ	RAC-gamma serine/threonine-protein kinase, protein kinase Akt-3, Protein kinase B gamma	M069–B10_078
Tor	target of rapamycin	M025–G4_020
	***Transcription regulators***	
Dmrt1	Sertoli cell marker, male sex determination	M057–E1_007
Blimp1	B lymphocyte maturation protein, repress somatic fates for germ cell fate in mouse	M054–B2_014
Bmi1, pcgf3	polycomb group ring finger 3	M090–E4_024
Klf4	Krüppel-like factor 4, essential for pluripotency	M046–D9_073
ATF4/Creb2	activating transcription factor 4/cAMP response element-binding protein 2	M031–D2_010
Pax6b	Paired box gene 6	M053—F2_006
Par3	leucine zipper kinase, essential for embryo polarity and blastomere identity	M076–D6_042
Phc2	polyhomeotic-like 2	M018–G2_004
Yin-Yang, YY1a	overexpression associated with unchecked cellular proliferation and resistance to apoptotic stimuli	M035–D3_025

**Table 5:** Gene markers for testicular cell types and spermatogenic stage in medaka testicular EST collection.

Gene name	Description	EST Clone ID
	***somatic cell marker***	
Dmrt1	Sertoli cell marker, male sex determination	M057–E1_007
Cyp45011b	cytochrome p450 11β, steroid synthesis, Sertoli marker	M020–D12_090
HSD3b	3-beta hydroxysteroid dehydrogenase, Leydig cell marker, steroid synthesis	M061–H12_082
LHR	luteinizing hormone receptor/gonadotropin receptor II, Leydig cell marker	M017–D7_057
Catenin α3	beta-catenin-binding protein, cell adhesion, myoid cell marker	M018–A12_096
	***Germ cell markers***	
Bruno2 = CUG2	CUG triplet repeat, RNA binding protein 2, translational repressor in fly germ cells	m012–G12_084
Gasz	germ cell-specific four ankyrin repeats and sterile-alpha motif and a basic leucine zipper	m013–E3_023
Gustavus	SPRY domain SOCS box protein, suppressor of cytokine signaling	M035–F5_037
mago nashi	proliferation-associated	M044–H1_001
Pelota	Polo-like kinase, germ cell development	M037–C10_076
Pili	Piwi-like, homologous to Drosophila Piwi	m014–E6_040
Pum1	Pumilio 1, homologous to Drosophila Pumilio	m006—C8_055
Pum2	pumilio 2, homologous to Drosophila Pumilio	M037–H6_034
Tdr9	tudor domain containing 9	M025–H12_082
Tdr11 = Snd1	staphylococcal nuclease&tudor domain1	M030–H9_065
	***Pre-meiosis***	
Cyclin E1	Cell cycle regulator, B spermatogonial marker	M062–G1_003
ALF	TFIIAa/b-like factor	M038–E5_039
PKA	cAMP-dependent protein kinase A, meiosis regulator	M049–C3_027
Clk2	CDC-like2 cell cycle regulator, B spermatogonia	M053–B4_030
	***Meiosis***	
Qkr/gld	quaking homolog, KH domain RNA binding protein for germline development and meiosis entry,	M089–G11_083
SCP1	synaptonemal complex protein 1, meiosis I prophase	M029–C7_059
Rad51	Eukaryotic homolog of RecA involved in DNA repair, meiosis I prophase	m015–H9_065
NME2	nucleoside diphosphate kinase B, oncogene/tumor suppressor	M034–G9_067
Mnd	meiotic nuclear division&recombination, meiosis I prophase	m008–G7_051
Chimerin	GTPase-activating protein, meiosis I prophase	m015–G2_004
	***Postmeiosis***	
Msap	meichroacidin-like sperm axonemal protein	M079–A4_032
Rsh	radial spokehead-like sperm axonemal protein	M037–C11_091

**Figure 2:**
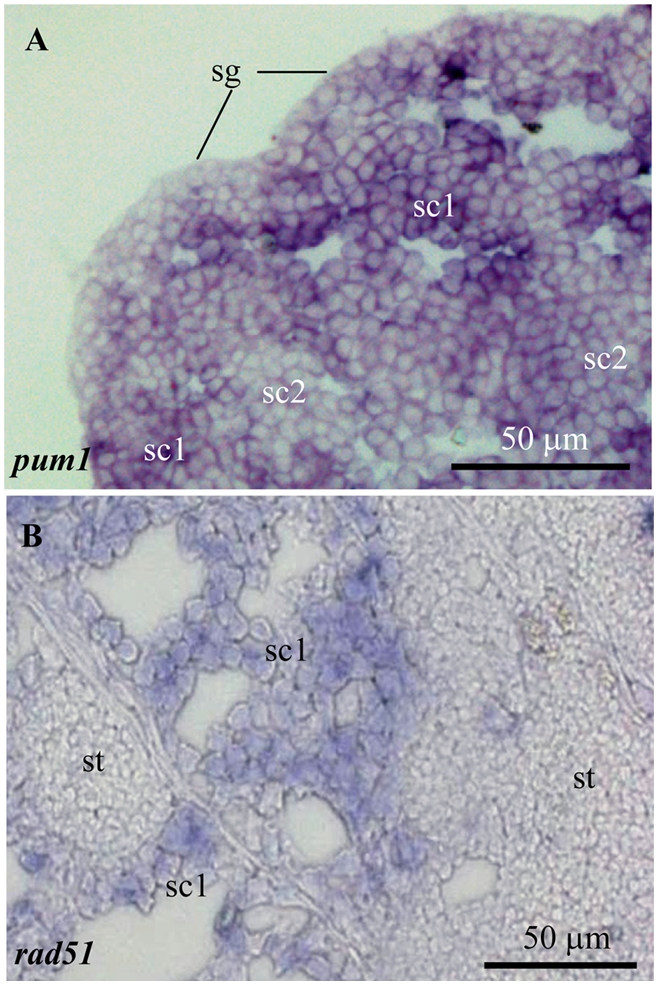
Analysis of *pum1* and *rad51* expression by in situ hybridization on medaka testicular sections. (A) *pum1* is expressed highly in the primary spermotocytes (sc1), moderately in the secondary spermatocytes (sc2) and weakly in the Spermatogonia (sg) at the periphery. (B) *rad51* is mainly expressed in the primary spermatocytes (sc1) but absent in spermatids (st).

To validate the genes obtained via cross-comparison analysis, expression patterns of two genes, namely *pum1* (germ cell marker) and *rad51* (meiosis marker), were examined via in situ hybridization in medaka testicular sections. The *pum1* gene is expressed weakly in Spermatogonia at the periphery, but highly in primary spermotocytes and moderately in secondary spermatocytes (Figure 2A). On the other hand, *rad51* is specifically expressed in the primary spermatocytes but absent in spermatids (Figure 2B).

### Counterparts of mouse X-chromosome encoded testis genes in medaka

A recent report identified 33 X-chromosomal genes whose expression are enriched in the mouse testis [30]. We cross-compared these 33 mouse genes with our medaka testis EST dataset and found that 13 of these are expressed in the medaka testis (Table 6), indicating that medaka and mammals share common features in testicular gene expression and possibly functions.

**Table 6:** Counterparts of 13 mouse X-chromosome encoded testis genes in the medaka testicular EST collection.

Mouse gene name	Genebank ID	Medaka EST clone ID	Homology (*e*-value)
Pabpc1l2	GC0XP072139	M063–H1_001	7E-80
MGC58426	237009[uid]	M054–D9_073	4E-48
Zfp161	gi|6678637	M068–A8_064	2E-34
Zxd	gi|158937319	m007–E1_007	2E-30
LOC278181	278181[uid]	M035–A4_032	5E-12
Tgif2lx	gi|23346541	M091–A10_080	5E-11
4933434C23Rik	71210[uid]	M079–B10_078	1E-9
Rhox	gi|115311558	M091–B4_030	4E-9
Ott	18422[uid]	M060–B12_094	2E-7
4930567H17Rik	619303[uid]	M084–F11_085	7E-6
LOC665542	665542[uid]	M061–H10_066	2E-4
4930527E24Rik	75140[uid]	M059–G3_019	4E-4
Srsx	100151772[uid]	M034–D8_058	5E-4

### Conclusion

We added 2607 new EST clusters (possibly unique genes) to the medaka EST collection and these new EST clusters/unigenes will be invaluable in assisting gene annotation once the medaka genome sequencing is completed. The wide range coverage of genes involved in diverse cellular activities and large number of novel sequences in our EST collection will not only consolidate our knowledge on known genes in testis [31]–[37] but also provide us rich resources to identify novel genes functioning during spermatogenesis. Although the 3640 EST clusters/unigenes were obtained from the testis majority of them will be house keeping genes (i.e also expressed in other organ/tissue) and only a fraction of these EST clusters/unigenes will be testis-enriched genes. Our long term goal is to carry out systemical screen for the testis-enriched genes. Take the advantage of the medaka fish we can study the functions and mechanisms of these testis-enriched genes in the future.

## Materials and Methods

### Construction of the normalized medaka testicular cDNA library

All procedures conducted with medaka fish are adhered to animal care guidelines (Guidelines on the Care and Use of Animals for Scientific Purposes) as outlined by the National Advisory Committee For Laboratory Animal Research in Singapore. Total RNA was extracted from pooled testes of adult medaka fish, using the Tri-Reagent according to the manufacturer's protocol (Molecular Research Centre Inc., USA). Total RNA was used for mRNA purification using mRNA purification kit (Qiagen, Germany). Both the 1^st^ and 2^nd^ stranded cDNA was synthesized using cDNA synthesis system (GibcoBRL, USA). Oligo-(dT)_20_-V (V = G, C, A) was used as primer for the 1^st^ strand cDNA synthesis. Two primers LLR1A (5′-gagatattagaattctactc) and LLR1B (complementary strand 5′-gagtagaattctaatat-3′) [17] were annealed at equal molar ratio and used as adaptor to ligate to the blunt-ended ds-cDNA and the ligated product was subjected to size selection. Total cDNA-adaptor ligated mix was loaded on an agarose gel (1%) for size fractioning and gel containing cDNA size between 0.5 kb and 2.0 kb was sliced out. This slice containing the size-selected cDNA was inserted into a pre-sliced slot in a fresh gel and electrophoresed in reverse current to concentrate the cDNA on the gel. Gel purified cDNA was amplified via PCR (denaturation at 94°C, 30 s; annealing using temperature gradient from 47°C to 50°C, 2 min; extension at 72°C, 3 min; 20 cycles) and the PCR product was pooled and concentrated for the first round denaturation/reassociation step (1 ug PCR product in 50 ul reassociation buffer containing 0.3 M Sodium Phosphate, 0.4 M EDTA, 0.04% SDS, pH 6.8). After denaturation at 100°C for 5 minutes, DNA was immediately transferred to 65°C for 24 hrs for reassociation and then quenched on ice. The yielded mixture of ss- and ds- cDNA was separated on a 1 cm Hydroxyapatite (Bio-Gel HTP gel # 130-0520, DNA-grade) jacketed column maintained at 65°C; using the AKTA FPLC system as described below. The reassociated DNA was diluted in 1 ml column equilibration buffer A (10 mM Sodium Phosphate, 0.1% SDS, pH 6.8, 65°C) and loaded onto the pre-equilibrated HA column. The column was washed with 3 CV (column volume) of buffer A, then eluted with a continuous gradient buffer from 0%–100% Buffer B (0.4 M Sodium Phosphate, 0.1% SDS pH 6.8, 65°C) over 10 CV, followed by 4 CV of buffer B to wash the column. ss-DNA eluted at ∼120 mM sodium phosphate and dsDNA at ∼300 mM sodium phosphate under these conditions. Fractions containing ssDNA were pooled and concentrated using Centricon-YM30 filter cartridge and the obtained ssDNA was used for the 2^nd^ round PCR. Two more rounds of normalization were performed and the final PCR products were digested with *EcoRI* and ligated to pre-digested pBluescript SK+ vector for library construction. Colony picking and bacteria culturing are as described previously [15].

### High throughput sequencing

Pasmid DNA was prepared in 96-well format using the conventional alkaline/SDS lysis method using robotics Biomek FX (Beckman) followed by ethanol precipitation [15]. Vector T3-primer was used to determine the EST sequence from each clone using either the Big Dye terminator cycle sequencing kit (Perkin Elmer) or DYEnamic ET terminator cycle sequencing kit (Amersham Pharmacia Biotech).

### Sequence Assembly

All sequences obtained were subjected to mass editing for vector and adaptor sequence clipping and elimination of low quality or short sequences using the pregap4 program in staden package (http://www.mrc-lmb.cam.ac.uk/pubseq/manual/pregap4_unix_toc.html). In total 7040 ESTs (7033 with reads >200 bp) were obtained after editing (Table1) and clustering using Tigr-Assembler (http://www.tigr.org/software/assembler) identified a total of 3641 unique clusters (Table S1).

### Sequence comparison against public database

The longest EST sequence in each of the 3641 unique clusters were retrieved and used as queries for BLASTN searches against the section Medaka UniGene (ftp://ftp.ncbi.nlm.nih.gov/repository/Unigene/) containing 17,307 unique clusters (released on February 2008). Sequences are considered identical if the blast E value is less than e^−50^ [38]. The longest EST in each of the 2607 unique clusters was translated into six frames and then compared to nr (ftp://ftp.ncbi.nlm.nih.gov/blast/db/nr). Only blast E value<e^−8^ were considered significant (Makabe et al. 2001).

### Gene ontology analysis

Gene information for the 1197 genes assigned with putative molecular and/or cellular function was analyzed manually and then classified based on their molecular and cellular functions.

### In situ hybridization and microscopy

In situ hybridization was performed essentially as described [39] by using the sense and antisense RNA probes derived from pum1 and rad51 clones, respectively. Observations and documentations were made under a Zeiss Axiovert invert microscope using a Zeiss Axiocam MRc digital camera.

## Supporting Information

Table S1 3641 unique medaka testis EST clusters.
(0.65 MB XLS)

Table S2 List of 1034 unique medaka testis EST clusters having hits in the medaka unigene database.
(0.26 MB XLS)

Table S3 List of 2057 unique medaka testis EST clusters having hit in the NCBI non-redundant database.
(0.42 MB XLS)

Table S4 Summary of ontology analysis of 1197 genes with known molecular/biochemical putative function
(0.21 MB XLS)

Table S5 List of 48 unigenes which have mRNA sequences in the unigene database.
(0.02 MB XLS)
